# Knowledge and practice of immediate new-born care among midwives in central zone public health facilities, Tigray, Ethiopia: cross sectional study

**DOI:** 10.1186/s13104-019-4532-5

**Published:** 2019-08-06

**Authors:** Tesfay Tsegay Gebru, Rajalakshmi Murugan, Alem Gebremariam Abrha, Mekonnen Haftom Goyteom

**Affiliations:** 1 Department of Nursing, Adigrat University, Adigrat, Ethiopia; 2 Department of Nursing, Addis Ababa University, Addis Ababa, Ethiopia; 3 Department of Public health, Adigrat University, Adigrat, Ethiopia

**Keywords:** Knowledge, Practice, Immediate new-born care, Tigray, Ethiopia

## Abstract

**Objective:**

The objective of this study was to assess knowledge and practice of immediate new-born care among midwives in central zone public health facilities Tigray region, 2016.

**Results:**

The mean age of the study participants was 34.1 years. Majority of the participants (83%) were diploma midwives. The score of knowledge of participants on immediate new-born care was 17.7% good and 25.2% poor. More than half (52.4%) of midwives practiced immediate new-born care. Midwives working in health center have 82% lower odds of new-born care compared to those working in hospital (p = 0.000, OR = 0.18 (0.07, 0.43).

**Electronic supplementary material:**

The online version of this article (10.1186/s13104-019-4532-5) contains supplementary material, which is available to authorized users.

## Introduction

New-born is completely tiny and powerless, and dependent on other [1]. Crying after delivery is an indicative of establishing active breathing [2]. Babies die after birth because they have difficulty of adapting to extra uterine life [3]. There are 20 countries with the highest neonatal mortality rates worldwide, out of this 16 are found in Africa including Ethiopia. One in 11 children in sub-Saharan Africa dies before celebrating their fifth years [4–6].

Even-though becoming new-born is not a disease, huge numbers of new-borns are dying immediately after birth [3]. Evidences showed that the day of birth is the riskiest time that is a child is 500 times more likely to die in the first day of life [4]. One million neonatal deaths occur on their day of birth [5, 7]. Knowledge and practice of health care providers is essential in improving the survival of new-born and reduces neonatal mortality and morbidity [8].

In Ethiopia 37 neonates died per 1000 live births [9]. Similarly in Tigray region, there are 32,098 live births. Even though midwives are more likely to attend labour and provide new-born care, 3515 (11%) neonates died at health facilities [10]. In Ethiopia specifically central zone of Tigray region health facilities there is no clear information regarding the knowledge and practice of midwives towards immediate newborn care. Therefore, it is crucial to assess the knowledge and practice of midwives towards immediate new-born care and provide well targeted intervention.

## Main text

### Methods

Institutional based cross-sectional study design was conducted from January to June, 2016 in central zone public health facilities of Tigray region. Central zone have 54 public health centers and 6 hospitals with 210 midwives working there.

A sample of 150 currently working midwives was included in the study. The sample size was determined using single population proportion formula. It was computed by considering 50% (p = 0.5) prevalence of knowledge and practice, 95% CI, and 5% margin of error. This resulted in 150 sample size after including 10% for non-response rate. Correction formula was used since the source population was less than the sampled population.

All public health facilities were included in the study. Final study subject was selected using convenience sampling method and midwives working in health facilities during the data collection time were included in the study.

Structured questionnaire was adapted with modification from different related literatures [1, 8, 11–13]. Observation checklist was adopted from save the children international [14]. The questionnaire and checklist were prepared first in English then translated to Tigrigna and retranslation to English. Questionnaire was pre-tested on 5% of the same source population other than the sampled population. Based on the pre-test, questions were revised, edited, and those found to be unclear or confusing were modified. Finally, structured closed ended Tigrigna version questionnaire was used for data collection. Moreover, checklist was used to assess immediate new care practice of the midwives.

Data was collected by face to face interview and non-participatory observations. Data collectors were five BSc midwives. They were trained for 2 days on the study instrument and data collection procedures. Additionally, two BSc Nurses, and assisted the data collectors.

#### Operational definition

##### Knowledge

Refers to the knowledge response of midwives to the structured questions on the steps of new-born care, that is good knowledge when they respond correctly to > 75% of the knowledge questions (> 8 steps), fair knowledge respond to 51–74% (5–8 steps) and poor knowledge respond to < 50% (< 5 steps) of the 10 cares given to immediately born baby.

##### Practice

Refers to the performance of midwives according to prepared checklist regarding new-born care. If the midwives performed the task at least 50% or above median (responded > 20 questions) of the steps in the checklists was considered as practiced, and not practiced if performed 50% or below median ≤ 20 questions) of the tasks in the checklists.

The collected data was entered into SPSS version 21.0. Variables with p-value less than 0.3 on bivariate analysis were entered into the multivariable analysis and adjusted odds ratio with 95% CI was used to ascertain the association between dependent and independent variables. The level of significance was taken at α < 0.05. Finally, result was presented in texts, and tables.

Ethical clearance and approval was obtained from research ethics committee of department of nursing and midwifery, Addis-Ababa University. Official cooperation letter was written from Tigray health bureau to each woreda health office and written permission was obtained from each respondents.

### Results

#### Participants’ socio-demographic characteristics

Totally 147 midwives were participated in the study which gives a response rate of 98%. Among the respondents 88 (59.9%) were from health center and 59 (40.1%) from hospitals. One hundred seventeen (79.6%) were females and 35 (23.8%) of the participants were in the age group of 25–29. Orthodox Christianity was the dominant religion consisting of 135 (91.8%). Majority (83.0%) of the respondents were diploma, 55 (37.4%) respondents had work experience of 1–5 years and 49.7% were married.

#### Participants knowledge on immediate newborn care

Participants responded that the advantage of early initiation of breastfeeding; 97 (66%) said, it prevents the newborn from hypoglycemia and 91 (61.9%) of respondents know the advantage of colostrum on preventing new-born baby from infection. Among all, 137 (93.2%) of respondents had knowledge on the appropriate time of initiation of breastfeeding for the newborn baby.

Most of the respondents know on placing the newborn on mother’s abdomen 122 (83%) immediately after delivery and 127 (86.4%) of midwives had knowledge of providing TTC eye ointment on both eyes (Additional file 1: Table S1). When the respondents asked about the immediate new-born complications, majority of them identified hypothermia, hypoxia and infection (Additional file 2: Table S2). Around 9 (6.15) of mothers wash babies before 24 h of delivery (Table 1).

**Table 1:** Knowledge of midwives on care given to immediately born baby in central zone, Tigray, Ethiopia, 2016

Variable	Frequency (N = 147)	Percent (%)
Knowledge of midwives on advantage of skin-to-skin contact		
Prevent hypothermia	64	55.8
Help baby stay warm	84	57.1
Bonding	40	27.2
Help expel placenta and uterine contraction	2	1.4
Knowledge of midwives on measures to be taken for baby unable to cry after delivery		
Suck the baby	136	92.5
Call a help and start resuscitation	105	71.4
Start cardio-pulmonary resuscitation	10	6.8
Kicking of the babies buttock	3	2.0
Oxygen administration	4	2.7
Knowledge on time of bathing for immediately born baby		
Before 24 h of delivery	9	6.1
After 24 h of delivery	110	74.8
I do not know	5	3.4
Counsels mother to wash at home after 24 h	23	15.6
Knowledge on the importance of providing eye ointment		
Prevent eye infection	57	38.8
Prevent blindness	12	8.2
Prevent conjunctivitis	21	14.3
Prevent from STI	23	15.7
Prevent gonorrhoea	2	1.4
Prevent syphilis	8	5.5
As prophylaxis	8	5.5
Prevent dryness of eye	1	0.7

The overall Knowledge of midwives on immediate newborn care was 17.7%, 57.1% and 25.2%, good, fair and poor knowledge respectively.

#### Participants practice of newborn care

Around 146 (99.3%) of respondents have prepared cord tie and clamp before delivery, but 98% of midwives did not prepared baby identification material (Table 2).

**Table 2:** Practice of midwives on immediate newborn care in central zone, Tigray, Ethiopia, 2016

Variable	Frequency N = 147	Percent (%)
Washes hands with soap and water, dried with a clean dry		
Perform task completely	36	24.5
Unable to perform task completely	111	75.5
Wipes the eyes and face when the head is delivered		
Perform task completely	94	63.9
Unable to perform task completely	53	36.1
Clean eyes immediately after birth with swab soaked in sterile water, using separate swab for each eye		
Perform task completely	44	29.9
Unable to perform task completely	103	70.1
Delivery surface covered with sterile dry towel		
Perform task completely	135	91.8
Unable to perform task completely	12	8.2
When baby not cried within 30 min of delivery, called help and prepared for steps of resuscitation		
Perform task completely	42	85.7
Unable to perform task completely	7	14.3
Use appropriate size of mask for resuscitation		
Perform task completely	45	91.8
Unable to perform task completely	4	8.2
Cord tie		
Perform task completely	84	57.1
Unable to perform task completely	63	42.9
Cord cut with sterile scissor or surgical blade		
Perform task completely	99	67.3
Unable to perform task completely	48	32.7

About 146 (99.3%) of participants in this study were not practiced to put baby identification bands on the wrist and ankle after delivery. Majority of the respondents, 145 (98.6%) have immediately dried the whole body of baby including the head and limbs but 14 (9.5%) of respondents have not removed wet cloth used to dry the baby. Most 145 (98.6%) of respondents were administered vitamin K to the new-born immediately.

Overall 52.4% of midwives practiced immediate newborn care appropriately.

#### Factors associated with immediate newborn care

Variables which have p-value less than or equal to 0.3 in Bivariate analysis were entered to multi-variable analysis. The multi-variable analysis result showed that work environment was significantly associated with practice of new-born care that is midwives working at health center were 82% lower odds of new-born care compared to those working in the hospitals (Table 3).

**Table 3:** Multivariable analysis of variables with practice of newborn care, among midwives working in central zone, Tigray, Ethiopia, 2016

Variables	Practiced new-born care		COR (95% CI)	AOR (95% CI)	p-value
	Yes, n (%)	No, n (%)			
Marital status					
Single	23 (48.9%)	24 (51.1%)	1	1	
Married	48 (65.8%)	25 (34.2%)	2.00 (0.94, 4.23)	1.90 (0.79, 4.58)	0.15
Divorced	2 (16.7%)	10 (83.3%)	0.20 (0.04, 1.05)	0.22 (0.04, 1.26)	0.09
Widowed	4 (26.7%)	11 (73.3%)	0.38 (0.10, 1.36)	0.40 (0.09, 1.73)	0.22
Religion					
Orthodox	74 (54.8%)	61 (45.2%)	1	1	0.036
Muslim	3 (25.0%)	9 (75.0%)	0.27 (0.07, 1.06)	0.19 (0.04, 0.89)	
Working environment					
Hospital	46 (78.0%)	13 (22.0%)	1	1	0.000
Health center	31 (35.2%)	57 (64.8%)	0.15 (0.07, 0.32)	0.18 (0.07, 0.43)	
Educational status					
Diploma	60 (49.2%)	62 (50.8%)	1	1	0.87
Degree	17 (68.0%)	8 (32.0%)	2.2 (0.88, 5.47)	1.10 (0.32, 3.72)	
Knowledge on newborn care					
Fair	43 (51.2%)	41 (48.8%)	1	1	
Good	16 (61.5%)	10 (38.5%)	1.52 (0.62, 3.75)	1.42 (0.48, 4.17)	0.52
Poor	18 (48.6%)	19 (51.4%)	0.90 (0.42, 1.96)	1.3 (0.51, 3.33)	0.57
Training on newborn care					
Yes	15 (71.4%)	6 (28.6%)	1	1	0.25
No	62 (49.2%)	64 (50.8%)	0.38 (0.14, 1.06)	0.48 (0.14, 1.65)	

### Discussion

According to this study even-though 85.7% of respondents had received in service training, participants had poor knowledge regarding care of new-born at birth (25.2%). This is in line with similar study done in Sudan [1]. This indicates that midwives in both study area have knowledge gap on immediate new-born care.

The knowledge of study participants on immediate new-born care of this survey were good (17.7%), fair (57.1%) and poor (25.2%). This is relatively lower than study done in Egypt [8]. This might be related to the educational status of respondent’s, there was presence of degree and speciality in the study done in Egypt.

About 99.3% of participants in this study were not practiced to put baby identification bands on the wrist and ankle after delivery. This is consistent with the study done in Khartoum, Sudan [1]. This will increases misshaping or exchange of babies in busy delivery room or time.

Practice of respondents towards immediate new-born care in this study were 52.4% which is relatively higher than the study done in Sudan [1]. This might be due to the descriptive statistics used at both study areas, that is the study done in Sudan had taken mean where as in this study practice was calculated from median.

This study result indicated that midwifes working at health center were 82% lower odds of new-born care compared to those working in the hospitals. This is different from the same study done by MAISHA program in Tanzania [12]. This might be due to difference in socio-demographic characteristics.

### Conclusion and recommendation

Based on the finding midwives had knowledge and practice gap on immediate new-born care. Statistically significant difference of new-born care was observed among the midwives working in the health center and hospital. In-service training and capacity building of the midwives related to knowledge and skill on newborn care is crucial. Special focus should provide to include immediate new-born care on pre-service training curriculum at college level.

## Limitation

Sampling procedure used for this study was convenience so it is limited to talk with this to the general population. The sample size used might not be enough to detect the statistical difference between the dependent and outcome variable. The nature of study design could not show seasonal variation and temporal relationship of cause and effect. Absence of similar literature in Ethiopia.

## Additional files

**Additional file 1: Table S1.** Knowledge on immediate newborn care of midwives at central zone Tigray region, Ethiopia, 2016.

**Additional file 2: Table S2.** Knowledge of midwives on complication of immediately born baby and preventive methods at central zone, Tigray region, Ethiopia, 2016.

## Data Availability

The datasets during and/or analyzed during the current study available from the corresponding author on reasonable request.

## Abbreviations

CI: confidence interval
SPSS: statistical package for social science
