# Supplementary material for: Knowledge and practice of immediate new-born care among midwives in central zone public health facilities, Tigray, Ethiopia: cross sectional study
Source: BMC Res Notes. 2019 Aug 6;12:487. doi: 10.1186/s13104-019-4532-5 (PMC6685261; doi:10.1186/s13104-019-4532-5)
Supplement: Supplementary file 1 — Additional file 1: Table S1. Knowledge on immediate newborn care of midwives at central zone Tigray region, Ethiopia, 2016. [file 13104_2019_4532_MOESM1_ESM.docx]

**Additional file 1:** Knowledge on immediate newborn care of midwives at central zone Tigray region, Ethiopia, 2016

| **Variable** | **Frequency (N=147)** | **Percent** |
| --- | --- | --- |
| ***Knowledge of midwives on care given to immediately born baby** | | |
| Put baby on to mother’s abdomen | 122 | 83% |
| Dry baby | 131 | 89.1% |
| Assessing breathing | 63 | 42.9% |
| Cord cutting and care | 137 | 93.2% |
| Eye care and applying TTC eye ointment | 127 | 86.4% |
| Early initiation of breast feeding | 98 | 66.6% |
| Skin-to-skin contact with mother | 91 | 61.9% |
| Giving vitamin K | 136 | 92.5% |
| Weigh baby | 81 | 55.1% |
| Record | 4 | 2.7 % |

* Each of the percentages does not add up to 100.0 because respondents could choose several responses
