# Supplementary material for: Knowledge and practice of immediate new-born care among midwives in central zone public health facilities, Tigray, Ethiopia: cross sectional study
Source: BMC Res Notes. 2019 Aug 6;12:487. doi: 10.1186/s13104-019-4532-5 (PMC6685261; doi:10.1186/s13104-019-4532-5)
Supplement: Supplementary file 2 — Additional file 2: Table S2. Knowledge of midwives on complication of immediately born baby and preventive methods at central zone, Tigray region, Ethiopia, 2016. [file 13104_2019_4532_MOESM2_ESM.docx]

**Additional file 2:** Knowledge of midwives on complication of immediately born baby and preventive methods at central zone, Tigray region, Ethiopia, 2016.

| **Variable** | **Frequency (N=147)** | **Percent** |
| --- | --- | --- |
| ***Knowledge of Midwives on complication of immediately born baby** | | |
| Hypothermia | 84 | 57.9% |
| Asphyxia | 121 | 83.4% |
| Infection | 70 | 48.3% |
| Hypoglycaemia | 10 | 6.9% |
| ***Knowledge of preventive methods for complication of newborn baby** | | |
| Put the baby on to mother’s abdomen | 53 | 36.6% |
| Assessing breathing | 42 | 29.0% |
| Clean cord cutting and care | 81 | 55.9% |
| Eye care and applying TTC eye ointment | 55 | 37.9% |
| Early initiation of breast feeding | 44 | 30.3% |
| Skin-to-skin contact with mother | 70 | 48.3% |
| Giving vitamin K | 26 | 17.9% |
| Weigh baby | 11 | 7.6% |
| Oxygen administration | 2 | 1.4% |
| Resuscitation | 7 | 4.8% |
| Suction | 37 | 25.5% |
| Use incubator/warmer | 1 | 0.9% |

* Each of the percentages does not add up to 100.0 because respondents could choose several responses
